# Caspase-1 is involved in the genesis of inflammatory hypernociception by contributing to peripheral IL-1β maturation

**DOI:** 10.1186/1744-8069-6-63

**Published:** 2010-10-04

**Authors:** Thiago M Cunha, Jhimmy Talbot, Larissa G Pinto, Silvio M Vieira, Guilherme R Souza, Ana T Guerrero, Fabiane Sonego, Waldiceu A Verri, Dario S Zamboni, Sergio H Ferreira, Fernando Q Cunha

**Affiliations:** 1 Department of Pharmacology, Faculty of Medicine of Ribeirão Preto, University of São Paulo, Av. Bandeirantes 3900, 14049-900, Ribeirao Preto, SP, Brazil; 2 Department of Cell Biology Faculty of Medicine of Ribeirão Preto, University of São Paulo, Av. Bandeirantes 3900, 14049-900, Ribeirao Preto, SP Brazil; 3 Universidade Estadual de Londrina, Centro de Ciências Biológicas, Departamento de Ciências Patológicas. Rodovia Celso Garcia Cid PR - 445 Km 379, 86051-970 - Londrina, PR Brasil

## Abstract

**Background:**

Caspase-1 is a cysteine protease responsible for the processing and secretion of IL-1β and IL-18, which are closely related to the induction of inflammation. However, limited evidence addresses the participation of caspase-1 in inflammatory pain. Here, we investigated the role of caspase-1 in inflammatory hypernociception (a decrease in the nociceptive threshold) using caspase-1 deficient mice (casp1-/-).

**Results:**

Mechanical inflammatory hypernociception was evaluated using an electronic version of the von Frey test. The production of cytokines, PGE_2 _and neutrophil migration were evaluated by ELISA, radioimmunoassay and myeloperoxidase activity, respectively. The interleukin (IL)-1β and cyclooxygenase (COX)-2 protein expression were evaluated by western blotting. The mechanical hypernociception induced by intraplantar injection of carrageenin, tumour necrosis factor (TNF)α and CXCL1/KC was reduced in casp1-/- mice compared with WT mice. However, the hypernociception induced by IL-1β and PGE_2 _did not differ in WT and casp1-/- mice. Carrageenin-induced TNF-α and CXCL1/KC production and neutrophil recruitment in the paws of WT mice were not different from casp1-/- mice, while the maturation of IL-1β was reduced in casp1-/- mice. Furthermore, carrageenin induced an increase in the expression of COX-2 and PGE_2 _production in the paw of WT mice, but was reduced in casp1-/- mice.

**Conclusion:**

These results suggest that caspase-1 plays a critical role in the cascade of events involved in the genesis of inflammatory hypernociception by promoting IL-1β maturation. Because caspase-1 is involved in the induction of COX-2 expression and PGE_2 _production, our data support the assertion that caspase-1 is a key target to control inflammatory pain.

## Background

Inflammatory hypernociception results mainly from the sensitisation of primary afferent neurons and is detected as a decrease of the nociceptive threshold in animal models [1]. It is induced by inflammatory mediators, such as prostaglandins and sympathetic amines, that directly sensitise peripheral nociceptive neurons [2-4]. The release of these direct-acting hyperalgesic mediators is generally preceded by a cascade of cytokines [5]. Recently, we demonstrated that inflammatory hypernociception in mice is mediated by a concomitant release of tumour necrosis factor alpha (TNFα) and keratinocyte-derived chemokine (CXCL1/KC). Both mediators stimulate the subsequent release of interleukin (IL)-1β that in turn induces prostanoid production [6]. CXCL1/KC also stimulates the sympathetic component of inflammatory hypernociception [6]. Another important event that mediates inflammatory hypernociception is neutrophil migration [7]. It has also been demonstrated that the hyperalgesic effect of cytokines depends on neutrophil recruitment that, in the last instance, seems to be important for the production of directly-acting hyperalgesic mediators such as prostaglandin E_2 _(PGE_2_) [7].

Caspase-1 (previously known as IL-1 converting enzyme -ICE) is a member of the caspase family of proteases and is responsible for the processing and secretion of IL-1β and IL-18, two cytokines that are critical for inflammation [8,9]. For instance, caspase-1-deficient mice (casp1-/-) have a defect in the maturation of pro-IL-1β and pro-IL-18 and are resistant to LPS-induced endotoxic shock [10]. Furthermore, the treatment of mice with a selective inhibitor of caspase-1 had reduced collagen-induced arthritis [11]. In this context, caspase-1 was considered an important target to control inflammatory diseases.

Although the action of caspase-1 in the maturation of IL-1β and IL-18 is an important step in the cascade of inflammatory events, there have been limited studies that investigate its role in the genesis of inflammatory pain [12,13]. For instance, a selective inhibitor of caspase-1 was found to inhibit yeast-induced hypernociception in rats [12]. Nonetheless, more conclusive evidence about the participation and the role of caspase-1 in inflammatory hypernociception is required. In the present study, we addressed the role of caspase-1 in carrageenin-induced mechanical inflammatory hypernociception using mice deficient in this enzyme. We focused mainly in the peripheral mechanisms involved in caspase-1 mediation of this inflammatory symptom. Our results indicate that caspase-1 plays a crucial role in genesis of inflammatory pain by promoting IL-1β maturation in the site of inflammation.

## Results

### Caspase-1-/- mice show reduced mechanical inflammatory hypernociception

**Figure 1:**
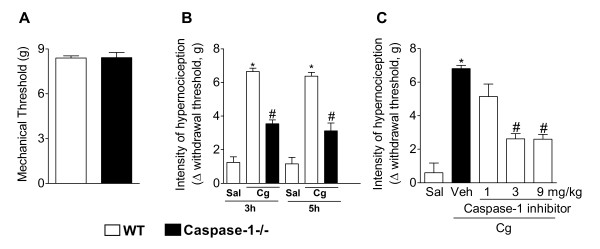
**The involvement of caspase-1 in mechanical inflammatory hypernociception**. **(A) **Mechanical nociceptive threshold of wild type and casp1-/- mice using the electronic von Frey. **(B) **Wild type or casp1-/- mice received an intraplantar injection of carrageenin (100 μg/paw). Mechanical hypernociception was evaluated 3 and 5 h after carrageenin injection. **(C) **Mice were pretreated with a caspase-1 inhibitor (YVAD-CMK, 1-9 mg/kg s.c. 30 min before) followed by intraplantar injection of carrageenin (100 μg/paw). Mechanical hypernociception was evaluated 3 h after carrageenin injection. Data are expressed as the mean ± S.E.M. of 5 animals per group. * indicates statistical significance compared to the saline injected group; # statistical significance compared to wild type or vehicle-treated group. *P *< 0.05, one-way ANOVA followed by the Bonferroni's test.

In the first set of experiments we evaluated the mechanical nociceptive threshold of WT and casp1-/- mice. Mechanical nociceptive thresholds of casp1-/- mice did not differ from WT littermates (Figure 1A). On the other hand, carrageenin-induced mechanical hypernociception was reduced in casp1-/- mice compared with WT mice. These differences were observed 3 and 5 h after stimuli injection (Figure 1B). The reduction in inflammatory hypernociception in casp1-/- mice was not accompanied by a reduction in carrageenin-induced paw oedema (WT/Saline 0.01 ± 0.003 mm^3^; WT/carrageenin 0,075 ± 0,007 mm^3 ^casp1-/-Saline 0,01 ± 0,004 mm^3^; casp1-/-/carrageenin 0,06 ± 0,005 mm^3^).

For a clinical perspective, we evaluated the effect of a selective inhibitor of caspase-1 (Ac-YVAD-CMK) on inflammatory hypernociception. It was observed that the pretreatment of mice with YVAD-CMK (1-9 mg/kg s.c.; 30 min before carrageenin injection), inhibited carrageenin-induced mechanical hypernociception in a dose-dependent manner (Figure 1C).

### Neither neutrophil nor pro-nociceptive cytokines (TNFα and CXCL1/KC) are involved in caspase-1 mediation of inflammatory hypernociception

**Figure 2:**
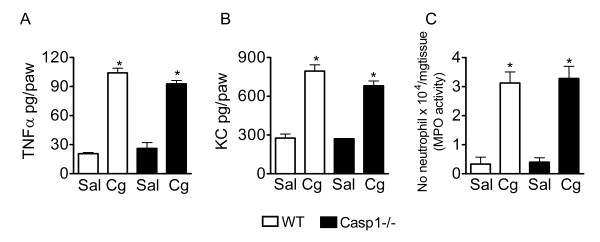
**Role of neutrophils and cytokines in caspase-1 mediation of inflammatory hypernociception**. **(A-B) **Wild type and casp1-/- mice received an intraplantar injection of carrageenin or saline. After 3 h, plantar tissue samples were removed and the levels of TNF-α and CXCL1/KC were determined by ELISA. **(C) **At 3 h after carrageenin injection, the activity of MPO was determined in the mice paw skin of wild type and casp1-/- mice as an index of neutrophil migration. Data are expressed as the mean ± S.E.M. of 5 animals per group. * indicates statistical significance compared to the saline-injected group. *P *< 0.05, one-way ANOVA followed by the Bonferroni's test.

Next, it was evaluated whether the reduction of inflammatory hypernociception in casp1-/- mice was associated with a reduction in the production of pro-nociceptive cytokines (TNFα or chemokines CXCL1/KC) or associated with a reduction in neutrophil migration. It was observed that the increase in the production of TNFα and CXCL1/KC after intraplantar injection of carrageenin in WT mice was not different from casp-1-/- mice (Figure 2A and B, respectively). Carrageenin-induced neutrophil migration was also not reduced in casp1-/- mice compared with WT mice (Figure 2C). In agreement with these findings, the doses of YVAD-CMK that inhibited carrageenin-induced mechanical hypernociception did not alter carrageenin-induced neutrophil migration (data not shown).

### Caspase-1 mediates TNFα and CXCL1/KC-induced mechanical hypernociception but is not induced by IL-1β or PGE_2_

**Figure 3:**
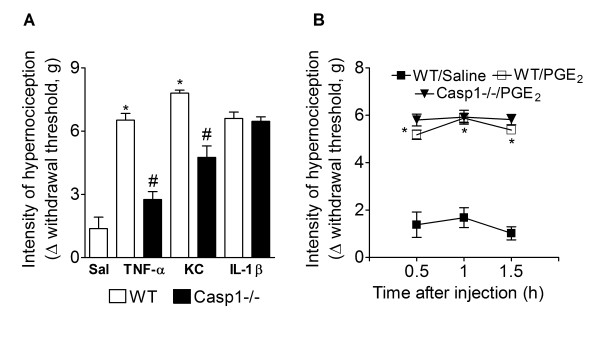
**Role of caspase-1 in the mechanical hypernociception induced by pro-nociceptive cytokines and PGE_2_**. **(A) **Wild type or casp1-/- mice received an intraplantar injection of TNF-α (100 pg/paw), CXCL1/KC (10 ng/paw or IL-1β (1 ng/paw). Mechanical hypernociception was evaluated 3 h after cytokines injection. **(B) **Wild type or casp1-/- mice received and intraplantar injection of PGE_2 _(100 ng/paw). Mechanical hypernociception was evaluated 0.5, 1.0, and 1.5 h after PGE_2 _injection. Data are expressed as the mean ± S.E.M. of 5 animals per group. * indicates statistical significance compared to the saline-injected group; # indicates statistical significance compared to wild type group. *P *< 0.05, one-way ANOVA followed by the Bonferroni's test.

Mechanical hypernociception induced by intraplantar injection of TNFα or CXCL1/KC was reduced in casp1-/- mice compared with WT mice (Figure 3A). On the other hand, the mechanical hypernociception induced by IL-1β or by PGE_2 _was not different in casp1-/- mice compared with WT mice (Figure 3A and 3B).

### IL-1β maturation in the inflammatory site, but not maturation of IL-18, accounts for caspase-1 mediation of inflammatory hypernociception

**Figure 4:**
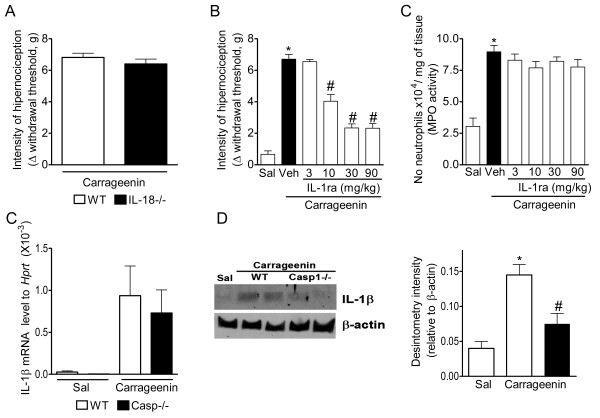
**IL-1β maturation, but not maturation of IL-18, is involved in caspase-1 mediation of inflammatory hypernociception**. **(A) **Wild type or IL-18-/- mice received an intraplantar injection of carrageenin (100 μg/paw). Mechanical hypernociception was evaluated 3 h after carrageenin injection. Mice were pretreated with IL-1ra (3-90 mg/kg, i.v. 15 min before carrageenin injection) followed by intraplantar injection of carrageenin (100 μg/paw). Mechanical hypernociception was evaluated 3 h after carrageenin injection. **(C) **After the determination of hypernociception, mice paw skins were removed and the activity of MPO was determined. **(D) **Wild type and casp1-/- mice received an intraplantar injection of carrageenin or saline. After 1.5 h, plantar tissue samples were removed and the level of pro-IL-1β mRNA was determined by real-time PCR. **(D) **Wild type and casp1-/- mice received an intraplantar injection of carrageenin or saline. After 3 h, plantar tissue samples were removed and the level of mature IL-1β (~19 kDa) was determined by western blot. The β-actin level was used as a control. Data are presented as representative blots. Densitometry of the pixel intensity of IL-1β bands relative to β-actin is present. Data are expressed as the mean ± S.E.M. of 5 animals per group. * indicates statistical significance compared to the saline-injected group; # indicates statistical significance compared to the vehicle-treated group or wild type mice group. *P *< 0.05, one-way ANOVA followed by the Bonferroni's test.

In the next part of this study, we tested the hypothesis that caspase-1 mediates inflammatory hypernociception by its action on IL-1β or IL-18 processing. Although carrageenin-induced mechanical hypernociception was not reduced in IL-18 null mice compared with WT mice (Figure 4A), the treatment of mice with IL-1ra (3-90 mg/kg, i.v. 15 min before carrageenin injection) reduced carrageenin-induced hypernociception in a dose-dependent manner (Figure 4B).On the other hand, IL-1ra treatment did not alter carrageenin-induced neutrophil migration toward mice paws (Figure 4C). The implication of IL-1β processing in the role of caspase-1 in inflammatory hypernociception was supported by the fact that while the increase in the expression of mRNA for pro-IL-1β induced in the mice paw by carrageenin was similar in WT and casp1-/- (Figure 4D), the expression of mature IL-1β (~19 kDa) was reduced in casp1-/- (Figure 4E). Densitometric analyses of the bands are present in Figure 4F.

### Peripheral COX-2 induction and PGE_2 _production depends on caspase-1

**Figure 5:**
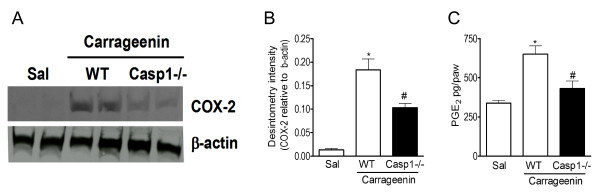
**Role of caspase-1 in the induction of COX-2 and prostaglandin production during carrageenin-induced inflammation**. **(A) **Wild type and casp1-/- mice received an intraplantar injection of carrageenin (100 μ/paw) or saline. After 3 h, plantar tissue samples were removed and the expression of COX-2 was determined by western blot. The β-actin level was used as a control. Data are presented as representative blots. **(B) **Densitometry of the pixel intensity of COX-2 bands relative to β-actin is present. **(C) **Wild type and casp1-/- mice received an intraplantar injection of carrageenin (100 μ/paw) or saline. After 3 h, plantar tissue samples were removed and the level of PGE_2 _was determined by RIA. Data are expressed as the mean ± S.E.M. of 5 animals per group. * indicates statistical significance compared to the saline-injected group; # indicates statistical significance compared to the wild type group. *P *< 0.05, one-way ANOVA followed by the Bonferroni's test.

Because it was previously demonstrated that the induction of COX-2 expression and the production of the direct-acting hypernociceptive mediator, PGE_2, _during inflammatory hypernociception is mediated by IL-1β [6]; we evaluated the contribution of caspase-1 in these events. Western blot analyses revealed that the increase in the COX-2 expression induced by carrageenin in WT mice is reduced in casp1-/- mice (Figure 5A). Densitometric analyses of the bands are present in Figure 5B. The reduction in COX-2 expression was also associated with a decrease in the production of PGE_2 _in casp1-/- mice compared with WT mice (Figure 5C).

## Discussion

Caspase-1, originally described as interleukin converting enzyme, is an intracellular enzyme that plays a role in the maturation of two important mediators of inflammation, IL-1β and IL-18 [14-16]. Recently, it was suggested that caspase-1 also metabolises another inflammatory mediator, IL-33; however, contrary to IL-1β and IL-18, it seems that caspase-1 action upon IL-33 produces an inactive form of this cytokine [17]. In the present study, we demonstrated that caspase-1 plays a crucial role in the cascade of events involved in the genesis of inflammatory hypernociception. Indeed, caspase-1 null mice present a reduction in carrageenin-induced hypernociception, and a selective inhibitor of caspase-1 presents an antinociceptive effect in this mice model. Our present results corroborate a previous finding of Elford *et al*. (1995) that showed that the treatment of rats with a selective inhibitor of caspase-1 reduced mechanical hypernociception in a model of yeast-induced inflammation [12]. Extending these observations, it was recently shown that caspase-1 mediates the induction of a complex regional pain syndrome that developed after tibia fracture in rats [18].

It is well known that the release of cytokines in the inflammatory site is involved in the induction of inflammatory hypernociception. In addition, it seems that these pro-nociceptive cytokines are released sequentially [6]. In this cascade, TNFα and chemokines, such as CXCL1/KC, are released earlier [6]. Investigating whether caspase-1 might account for the release of these two cytokines, we detected that the levels of TNFα and CXCL1/KC in carrageenin-inflamed mice paws are similar in WT and casp1-/- mice, suggesting that the caspase-1 pro-nociceptive role is downstream of TNFα and CXCL1/KC. Our results also refute that the pro-nociceptive role of caspase-1 in carrageenin inflammation is dependent on its capacity to process IL-18. This conclusion is based in the fact that IL-18 null mice present similar hypernociception after carrageenin paw injection compared with WT mice. Nonetheless, the involvement of caspase-1 in complex regional pain syndrome triggered by tibia fracture seems to be dependent on IL-18 processing [18]. Moreover, IL-18 also plays a role in the genesis of hypernociception in a model of immune inflammation induced by ovalbumin in previously immunised mice, and in this model caspase-1 might be responsible for IL-18 processing [19]. We also recently showed that besides cytokines, the recruitment of neutrophils to inflammatory sites mediates carrageenin-induced hypernociception [7]. However, it is also seems that the caspase-1 pro-nociceptive role is not related to neutrophils, because neutrophil accumulation in the mice paws of casp1-/- is similar to WT mice.

TNFα and CXCL-1/KC hypernociceptive roles in carrageenin inflammation were demonstrated to be, at least in part, dependent on the production of IL-1β [6]. This fact, together with our present observation that casp1-/- mice present reduced hypernociception induced by TNF-α and CXCL1/KC but not by IL-1β and PGE_2, _is strongly suggestive that caspase-1 could be mediating inflammatory hypernociception through its product, mature IL-1β. Indeed, we detected that while the mRNA for pro-IL-1β increased at the same levels in casp1-/- and WT mice after carrageenin injection, there was a reduction in the mature form of IL-1β in casp-1-/- mice. This suggestion was supported by the observation that IL-1ra treatment reduced carrageenin-induced hypernociception. Corroborating the idea that IL-1β processing is the mechanism by which caspase-1 mediates inflammatory hypernociception, it was observed that IL-1ra treatment did not alter the neutrophil migration induced by carrageenin similarly observed in casp1-/- mice. It is somehow striking because IL-1β hypernociceptive effect is dependent on neutrophil migration [7]. At this moment, we have the hypothesis that that in carrageenin-induced inflammation neutrophils are recruited mainly by TNFα and CXCR2 ligands (CXCL1/KC in mice or CINC-1 in rats) and they are activated in the site of inflammation by IL-1β that in turn induced the expression of COX-2 and the production of PGE_2_.

Although casp1-/- mice present a reduction in the expression of the mature form of IL-1β during carrageenin-induced paw inflammation, these mice still present the residual production of active IL-1β, suggesting that alternative mechanisms might trigger the induction of mature IL-1β in this model. For instance, there is evidence that pro-IL-1β can be cleaved by other proteases in addition to caspase-1, including elastase, proteinase 3 matrix metalloprotease 9 (MMP9), which are produced by neutrophils recruited to sites of tissue damage [20-23]. In this context, we have recently shown that MMP9 mediates hypernociception that developed during antigen-induced arthritis, a model in which IL-1β also plays a role [24].

Besides peripheral participation in the development of inflammatory hypernociception, we could not disregard the fact that caspase-1 might be involved by playing a central role in the response. In fact, activation of caspase-1 in the spinal cord has been associated with an increase of central IL-1β production that promotes COX-2 dependent inflammatory hypernociception [13]. In the periphery, the pro-nociceptive effect of IL-1β was also mediated by cyclooxygenase-derived PGE_2 _because its effect was inhibited by treatment with indomethacin [6,25]. Therefore, the observation that COX-2 expression and PGE_2 _production are reduced in casp1-/- mice challenged with carrageenin corroborates the importance of IL-1β in these processes. Contrary to these results, it was shown that although spinal glial-derived IL-1β is fundamental for the development of neuropathic pain after peripheral nerve injury, caspase-1 is not involved in this process [23]. It was clearly demonstrated that, in this model, MMP-9 and MMP-2 are the enzymes responsible for IL-1β maturation playing a central role in neuropathic pain induction [23].

One question that emerges from these results is how caspase-1 is activated during carrageenin-induced paw inflammation. Regarding the mechanisms that trigger caspase-1 activation in the inflammatory process, there is now a large body of recent evidence showing that they are dependent on the assembly of cytosolic multiprotein complexes known as inflammasomes [26,27]. Inflammasomes are formed by self-oligomerising scaffold proteins belonging to the NOD-like receptor family. There are, at least, four different inflammasomes: NALP1/NLRP1, NALP3/NLRP3, IPAF/NLRC4 and the HIN-200 family member, AIM2 [26]. These molecules self-oligomerise after stimuli recognition and form high-molecular weight complexes that trigger caspase-1 autoactivation. Therefore, an interesting question is: which inflammasome is activated during carrageenin inflammation? In our knowledge, there is no study that addresses this issue. However, one can predict the involvement of the NALP3 inflammasome. This suggestion is based on the following indirect evidence: a) NALP3-containing inflammasomes that activate caspase-1 generally depend on stimulation of P2X7 [28]; b) P2X7 mediates carrageenin-induced inflammatory hypernociception [29,30]; and c) P2X7 mediation of inflammatory hypernociception depends on stimulation of IL-1β because the antinociceptive effect of a selective P2X7 receptor antagonist is lost in IL-1 knockout mice [30]. Collectively, these data suggest that NALP3 is the inflammasome that triggers caspase-1 activation in the mediation of carrageenin-induced inflammatory hypernociception, yet other inflammasomes could be also involved. For instance, the NALP1 inflammasome is required for caspase-1 activation and mediates the complex regional pain syndrome that developed after tibia fracture in rats [18]. Therefore, additional studies using, for example, NALP3 null mice are required to solve this question.

In summary, the present study presents evidence that caspase-1 is involved in the genesis of inflammatory hypernociception. The participation of caspase-1 in this inflammatory symptom was not associated with the production of pro-inflammatory cytokines (TNFα, CXCL1/KC or IL-18) and recruitment of neutrophils, but was associated with the maturation of IL-1β in the inflammatory site. Caspase-1 seems to also be involved in the induction of COX-2 and consequently in the production of the directly-acting hypernociceptive mediator, PGE_2_. Together, these results added new information about the physiopathology of inflammatory pain. In conclusion, it is plausible to suggest that caspase-1 constitutes a real target to control inflammatory pain.

## Methods

### Animals

The experiments were performed on male C57BL/6 mice (wild type, WT, 20-25 g) and caspase-1 deficient mice (casp1-/-). Breeding pairs of mice with targeted disruption of the caspase-1 gene were back-crossed with C57BL/6 for 8 generations. BALB/C mice were used as a control when the experiment was conducted in IL-18 null mice (IL-18-/-). They were housed in the animal care facility of the School of Medicine of Ribeirao Preto and taken to the testing room at least one hour before the experiments. Food and water were available *ad libitum*. All behavioural tests were performed between 9:00 AM and 5:00 PM, and the animals were used only once. Animal care and handling procedures were in accordance with the guidelines of the International Association for the Study of Pain (IASP) on the use of animals in pain research. All efforts were made to minimise the number of animals used and their discomfort.

### Drugs

The drugs used in this study were: prostaglandin E_2 _(PGE_2_) (from Sigma, St. Louis, MO), YVAD-CMK (Calbiochem, San Diego, CA, USA), TNFα, IL-1β and IL-1 receptor antagonist (IL-1ra) (NIBSC, South Mimms, Hertfordshire, U.K.), CXCL1/KC (PeproTech, Rocky Hill, NJ), and carrageenin (FMC, Philadelphia). A stock solution of PGE_2 _(1 μg/μL) was prepared in 10% ethanol, and dilutions were made in 0.9% NaCl (saline); the final concentration of ethanol was 1%. Other drugs were diluted in sterile saline.

### Mechanical nociceptive paw test

Mechanical hypernociception was tested in mice as previously reported [31]. In a quiet room, mice were placed in acrylic cages (12 × 10 × 17 cm) with wire grid floors 15-30 min before the start of testing. The test consisted of evoking a hindpaw flexion reflex with a handheld force transducer (electronic aesthesiometer, IITC Life Science, Woodland Hills, CA) adapted with a 0.5 mm^2 ^polypropylene tip. The investigator was trained to apply the tip perpendicularly to the central area of the plantar hindpaw with a gradual increase in pressure. The gradual increase in pressure was manually performed in blinded experiments. The upper limit pressure was 15 g. The end-point was characterised by the removal of the paw followed by clear flinching movements. After paw withdrawal, the intensity of the pressure was automatically recorded, and the final value for the response was obtained by averaging three measurements. The animals were tested before and after treatments. The results are expressed by the delta (Δ) withdrawal threshold (in g) calculated by subtracting the zero-time mean measurements from the mean measurements at the indicated times after drug or solvent (control) injections. The withdrawal threshold was 8.9 ± 0.2 g (mean ± S.E.M.; n = 30) before injection of the solvent or hypernociceptive agents.

### Cytokine measurements

At indicated times after the injection of inflammatory stimuli, the animals were terminally anesthetised, and the skin tissues of the plantar region were removed from the injected and control paws (saline and naïve). The samples were triturated and homogenized in 500 μl of the appropriate buffer (phosphate-buffered saline containing 0.05% Tween 20, 0.1 mM phenylmethylsulphonyl fluoride, 0.1 mM benzethonium chloride, 10 mM EDTA and 20 kallikrein international units of aprotinin A) followed by a centrifugation of 10 min at 2000 g. The supernatants were used to determine the levels of TNF-α and CXCL1/KC as described previously [32] by enzyme-linked immunosorbent assays (ELISA). Briefly, microtiter plates were coated overnight at 4°C with an immunoaffinity-purified polyclonal sheep antibody against TNFα (2 μg/mL) or CXCL1/KC (1 μg/mL). After blocking the plates, recombinant murine TNFα or CXCL1/KC standards at various dilutions and the samples were added in duplicate and incubated overnight at 4°C. Rabbit biotinylated immunoaffinity-purified antibody anti- anti-TNFα (1:500) or CXCL1/KC (0.2 μg/mL) were added, followed by incubation at room temperature for 1 h. Finally, 50 μL of avidin-HRP (1:5000 dilution; DAKO A/S, Denmark) was added to each well, and after 30 min the plates were washed and the colour reagent OPD (200 μg/well; Sigma) was added. After 15 min, the reaction was stopped with 1 M H_2_SO_4 _and the optical density (O.D.) reading at 490 nm was taken. The results are expressed as picograms (pg) of each cytokine per paw.

### Neutrophil migration to the plantar tissue

Neutrophil migration to the hind paw plantar tissues of mice was evaluated using a myeloperoxidase (MPO) kinetic-colorimetric assay as previously described [33,34]. Samples of subcutaneous plantar tissue were collected in 50 mM K_2_HPO_4 _buffer (pH 6.0) containing 0.5% hexadecyltrimethylammonium bromide (HTAB) and kept at -80°C until use. Samples were homogenised using a Polytron (PT3100), centrifuged at 16,100 *g *for 4 min, and the resulting supernatant was assayed for MPO activity spectrophotometrically at 450 nm (Spectra max), with three readings in 1 min. The MPO activity of samples was compared to a standard curve of neutrophils. Briefly, 10 μL of sample was mixed with 200 μL of 50 mM phosphate buffer, pH 6.0, containing 0.167 mg/mL *O*-dianisidine dihydrochloride and 0.0005% hydrogen peroxide. The results are presented as MPO activity (number of neutrophils per mg of tissue).

### RNA extraction and Real-Time PCR

At 1.5 h after intraplantar (i.pl.) injection of carrageenin or saline, mice were terminally anesthetised, and skin tissues were removed from the plantar region of paws. The samples were homogenised in 1 mL of TRIzol (Invitrogen, Carlsbad, CA) and total RNA was extracted following the manufacturer's instructions. The purity of total RNA was measured with a spectrophotometer and the wavelength absorption ratio (260/280 nm) was between 1.8 and 2.0 for all preparations. Reverse transcription of total RNA to cDNA was carried out with a reverse transcription reaction (Superscript II, Gibco Life Technologies, Grand Island, NY, USA). Real-time PCR was performed using primers specific for the mouse gene pro-IL-1β and for the mouse housekeeping gene hypoxanthine guanine phosphoribosyl transferase (Hprt). Reactions were conducted on the ABI Prism 7500 Sequence Detection System using the SYBR-green fluorescence system (Applied Biosystems, Warrington, UK). The data were analysed with the 2^−ΔΔCt ^method as described previously [35] and they are expressed relative to samples collected in the saline group of animals. Primer pairs for mouse *Hprt *and pro-IL-1β were as follows:

pro-IL-1β fwd: 5'-GCTGCTTCCAAACCTTTGAC-3'

pro-IL-1β rev: 5'-AGCTTCTCCACAGCCACAAT-3'

Hprt fwd: 5'-GCCCCAAAATGGTTAAGGTT- 3'

Hprt rev: 5'-CAAGGGCATATCCAACAACA- 3'

### Western blot analysis

At 3 h after i.pl. injection of carrageenin or saline, mice were terminally anesthetised, and skin tissues were removed from the plantar region of paws. The samples were homogenised and the expression of cyclooxygenase (COX-2; ~72 kDa) or the mature form of IL-1β (~19 kDa) were evaluated using western blot analyses. Briefly, samples were homogenised in a lysis buffer containing a mixture of proteinase inhibitors (Tris-HCl 50 mM, pH 7.4; NP-40 1%; Na-deoxycholate 0.25%; NaCl 150 mM; EDTA 1 mM; PMSF 1 mM; Aprotinin, leupeptin and pepstatin 1 μg/mL each). Proteins were separated by SDS-polyacrylamide gel electrophoresis (SDS-PAGE-12%) and transblotted onto nitrocellulose membranes (Amersham Pharmacia Biotech, Little Chalfont, UK). The membranes were blocked with 7% dry milk (overnight) and incubated overnight at 4°C with a rabbit polyclonal antibody against COX-2 (1:400; Cayman, Ann Arbor, Michigan, USA) or IL-1β (1:200, Santa Cruz, CA, USA). After these procedures, the membranes were washed and then incubated for 1 h at room temperature with an HRP-conjugated secondary antibody (1:20000; Jackson ImmunoResearch, PA, USA). The blots were visualised in ECL solution (Amersham Pharmacia Biotech) for 2 min and exposed onto sheets of Hyperfilm (Amersham Pharmacia Biotech) for 2-20 min. A β-actin (1:2000; AbCam, Cambridge, MA, USA) antibody was used for loading controls. A total of ten western blot runs were performed and transferred to eight nitrocellulose membranes with proteins of the saline group and carrageenin injection group in WT and casp-1-/- mice. Films were scanned into Image Quant 5.2 for analysis. A computer-based imaging system (Gel-Pro Analyzer) was used to measure the intensity of the optical density of the ~70 kDa and ~19 kDa bands that represent the molecular weight of COX-2 and the mature form of IL-1β proteins.

### Measurement of PGE_2 _in paw skin

The plantar tissues were collected 3 h after intraplantar injection of carrageenin (100 μg/paw) or saline as described above. This time point is the peak of carrageenin-induced PGE_2 _production in the mice paw (data not shown). The paws were injected with indomethacin (50 μg/paw) 10 min before tissue retrieval to block PGE_2 _production during tissue processing. The PGE_2 _was extracted from plantar tissue and determined by radioimmunoassay [7]. Briefly, the plantar tissue samples were homogenized in a mixture of 3.0 ml of extraction solvent (isopropanol/ethyl acetate/0.1 N HCl, 3:3:1) and 3.0 ml of distilled water. Also, the solution contained 20 μg/ml of indomethacin. Homogenates were centrifuged at 1,500 *g *for 10 min at 4°C. The organic phase was aspirated and evaporated to dryness in a centrifugal evaporator. The pellet was reconstituted in 500 μl of 0.1 M phosphate buffer (pH 7.4) containing 0.8% sodium azide and 0.1% gelatin. Concentration of PGE_2 _in these samples was then measured by RIA by using a commercially available kit. The results are expressed as picograms of PGE_2 _per paw.

### Paw oedema test

The volume of the mice paw was measured with a plesthismometer (Ugo Basil, Italy) before (Vo) the intraplantar stimulus with carrageenan and 3 h after (VT), as described previously [34]. The amount of paw swelling was determined for each mouse and the difference between VT and Vo was taken as the oedema value (oedema mm^3^/paw).

### Statistical analysis

All results are presented as means ± S.E.M. The experiments were repeated at least twice. The "n" in the legends refers to the number of mice used in the experimental group of each experiment. The differences between the experimental groups were compared by ANOVA (one-way) and individual comparisons were subsequently made with Tukey's post hoc test. The level of significance was set at *P *< 0.05.

## List of Abbreviations

KC/CXCL1: keratinocyte-derived chemokine; TNF: tumour necrosis factor; TNFR: tumour necrosis factor receptor; PGE_2_: prostaglandin E_2_: IL, interleukin; casp1-/-: caspase-1-deficient mice; i.pl.: intraplantar; MPO: myeloperoxidase; Hprt: hypoxanthine guanine phosphoribosyl transferase; WT: wild type; COX-2: cyclooxygenase-2; IL-1ra: IL-1 receptor antagonist; MMP: matrix metalloprotease.

## Competing interests

The authors declare that they have no competing interests.

## Authors' contributions

TMC, LGP, GRS, ATG and WAV carried out the behavioral studies, TMC, JT, SMV and FS carried out molecular and biochemical studies, TMC, DSZ, SHF and FQC conceived of and designed the study and TMC wrote the manuscript. All authors read and approved the final manuscript.
